# Unveiling the Role of β-Glucosidase Genes in *Bletilla striata*’s Secondary Metabolism: A Genome-Wide Analysis

**DOI:** 10.3390/ijms252313191

**Published:** 2024-12-08

**Authors:** Mengwei Xu, Hongwei Li, Hongyuan Luo, Jingyi Liu, Kunqian Li, Qingqing Li, Ning Yang, Delin Xu

**Affiliations:** 1 Department of Medical Instrumental Analysis, Zunyi Medical University, Zunyi 563099, China; biologyxmw@126.com (M.X.); 15285105593@163.com (J.L.); mfkunqian@163.com (K.L.); qingqingli2023@163.com (Q.L.); 2 Department of Cell Biology, Zunyi Medical University, Zunyi 563099, China; 18323158371@163.com (H.L.); hongyuanluo@126.com (H.L.)

**Keywords:** *Bletilla striata*, β-glucosidase, phylogenetic metabolites, genome-wide analysis, phylogenetic analysis, gene expression profiles, mass spectrometry

## Abstract

β-glucosidases (BGLUs) are abundant enzymes in plants that play pivotal roles in cell wall modification, hormone signal transduction, secondary metabolism, defense against herbivores, and volatile compound release. *Bletilla striata*, a perennial herb revered for its therapeutic properties, lacks a comprehensive analysis of its *BGLU* gene family despite the critical role these genes play in plant secondary metabolism. This study aims to perform a genome-wide analysis of the *BGLU* gene family in *B. striata* (*BsBGLU*) to elucidate their functions and regulatory mechanisms in secondary metabolite biosynthesis. We conducted a genome-wide screening to identify *BsBGLU*, followed by phylogenetic analysis to classify these genes into groups. Sequence characteristics were analyzed to predict functional roles. Simple sequence repeat (SSR) markers were examined to assess conservation and polymorphism among different landraces. Expression profiles of *BsBGLUs* were evaluated under sodium acetate and salicylic acid elicitor treatments and across different tissues. The accumulation of phylogenetic metabolites in different treatments and tissues was also analyzed by HPLC and LCMS detection to explore the correlation between gene expression and metabolite accumulation. A total of 23 *BsBGLU* genes were identified and classified into eight distinct groups. Sequence analysis suggested diverse functions related to hormone responses, secondary metabolism, and stress resistance. *BsBGLUs* with SSR sequences were conserved yet showed polymorphism among different *B. striata* landraces. Under elicitor treatments, expression profiling revealed that *BsBGLUs* significantly modulate the synthesis of secondary metabolites such as dactylorhin A and militarine. Tissue-specific expression analysis indicated that *BsBGLU15* and *BsBGLU28* were highly expressed in tubers compared to other tissues, suggesting their central role and a potential negative regulatory effect in metabolite accumulation. The elicitor NaAc can regulate metabolite synthesis by modulating the expression of *BsBGLUs*. The *BsBGLU* gene family in *B. striata* is integral to the modulation of secondary metabolite biosynthesis and accumulation and can respond to elicitors to promote the synthesis of militarine. These findings provide a theoretical foundation for the further exploration of *BsBGLU* gene functions and their regulatory mechanisms, advancing the production of medicinally active compounds in *B. striata.*

## 1. Introduction

*Bletilla striata* (Thunb.) Rchb.f., a perennial medicinal herb of the Orchidaceae family, is renowned for its significant pharmacological properties, including hemostatic, anti-inflammatory, and anti-ulcerative effects [1]. These therapeutic activities are attributed to its core active compounds, such as militarine [(1,4-bis [4-(β-D-glucopyranosyloxy) benzyl]-2-isobutylmalate)], dactylorhin A, 9,10-dihydrophenanthrene, and other secondary metabolites [2]. Notably, militarine, a β-glucoside monomer with various biological activities, plays a crucial role in the medicinal efficacy of *B. striata*, exhibiting neuroprotective, hemostatic, and anti-ulcer properties [3,4]. However, insufficient understanding of the biosynthetic mechanisms of secondary metabolites hinders the analysis of their pharmacological effects and the development of new medicines. β-glucosidases (BGLUs, EC 3.2.1.21) belong to the glycoside hydrolase (GH) family, which comprises 166 subfamilies [5]. They catalyze the hydrolysis of terminal non-reducing β-D-glucose residues in glycosyl esters, oligosaccharides, and glycosides, releasing β-D-glucose [6]. Among the GH families, GH1 is the largest subfamily, and most plant BGLUs identified to date belong to this group [7]. These BGLUs are implicated in various biological processes related to plant growth and development, such as cell wall modification, lignification, and secondary metabolism [8,9]. Several studies have demonstrated the crucial roles of BGLUs in plant hormone signaling and stress responses. For instance, *PsBGLU12* and *PsBGLU24* are involved in endogenous hormone signal transduction and glucose and starch metabolism in *Papaver somniferum* [10]. In *Oryza sativa*, BGLUs act as primary modulators in response to environmental pollutants and are associated with auxin (IAA) and abscisic acid (ABA) signaling, affecting seed germination, root elongation, and drought resistance [11,12]. Collectively, these investigations highlight the pivotal role of BGLUs in plant growth, development, and stress adaptation. Despite the importance of BGLUs in plant secondary metabolism and the therapeutic significance of *B. striata*, there is a lack of comprehensive genome-wide analysis of the *BGLU* gene family in this species. Our previous transcriptome studies indicated that BGLUs are key enzymes in the accumulation of polysaccharides and phenolic acid metabolites under plant hormone treatments [13], and we explored the regulatory mechanisms of key enzymes involved in metabolite accumulation upon elicitor treatments with salicylic acid (SA) and sodium acetate (NaAc) [14]. Therefore, screening BsBGLU in the *B. striata* genome and elucidating their roles in secondary metabolite biosynthesis are essential for advancing our understanding of their functions.

In this study, we performed a comprehensive genome-wide analysis of the BsBGLU in *B. striata*. In the previous study, we employed transcriptomics and metabolomics sequencing techniques to identify differentially expressed genes and differentially accumulated metabolites in suspension-cultured cells. Correlation analysis revealed a significant positive correlation between multiple *BGLU* genes and the content of militarine [2]. Consequently, we have selected all *BGLU* sequences from the transcriptome data as the focal point of our research for further exploration. Using multiple sequence alignment, we identified 23 candidate GH1 BsBGLU members and analyzed their sequence characteristics, phylogenetic relationships, conserved motifs, gene structures, cis-elements, and expression patterns under stress conditions. In addition, we identified sequences with strong correlations based on the comprehensive analysis results and further investigated their relationship with militarine accumulation by assessing their expression levels in four different tissues, as well as measuring the corresponding accumulation of metabolites. This research provides valuable insights into the evolution of the BGLU family in orchids and lays a foundation for further functional studies and molecular breeding aimed at enhancing the production of medicinally active compounds in *B. striata.*

## 2. Results

### 2.1. Analysis of Nucleotide and Amino Acid Sequence Characteristics

#### 2.1.1. Identification and Physicochemical Properties of BsBGLU Amino Acid Sequence

A total of 31 unique BsBGLU sequences were identified from the *B. striata* transcriptome database using BLAST and Pfam analyses, and these sequences were designated BsBGLU1 to BsBGLU31. Upon careful examination, 23 of these sequences were confirmed to contain the conserved glycoside hydrolase family 1 (GH1) domain (PF00232) and were considered bona fide members of the *BsBGLU* gene family.

Physicochemical properties of protein analysis showed that the amino acid lengths of the BsBGLU proteins ranged from 52 to 516 residues, with predicted molecular weights between 6.02 kDa and 58.85 kDa (Table 1). The grand average of hydropathicity (GRAVY) values for all BsBGLU proteins were negative, indicating that they are hydrophilic in nature and likely soluble within the cellular environment. Secondary structure predictions revealed that higher proportion of α-helices compared to β-sheets suggests structural stability, allowing BsBGLU proteins to maintain the precise spatial conformation necessary for biological activity.

**Figure 1:**
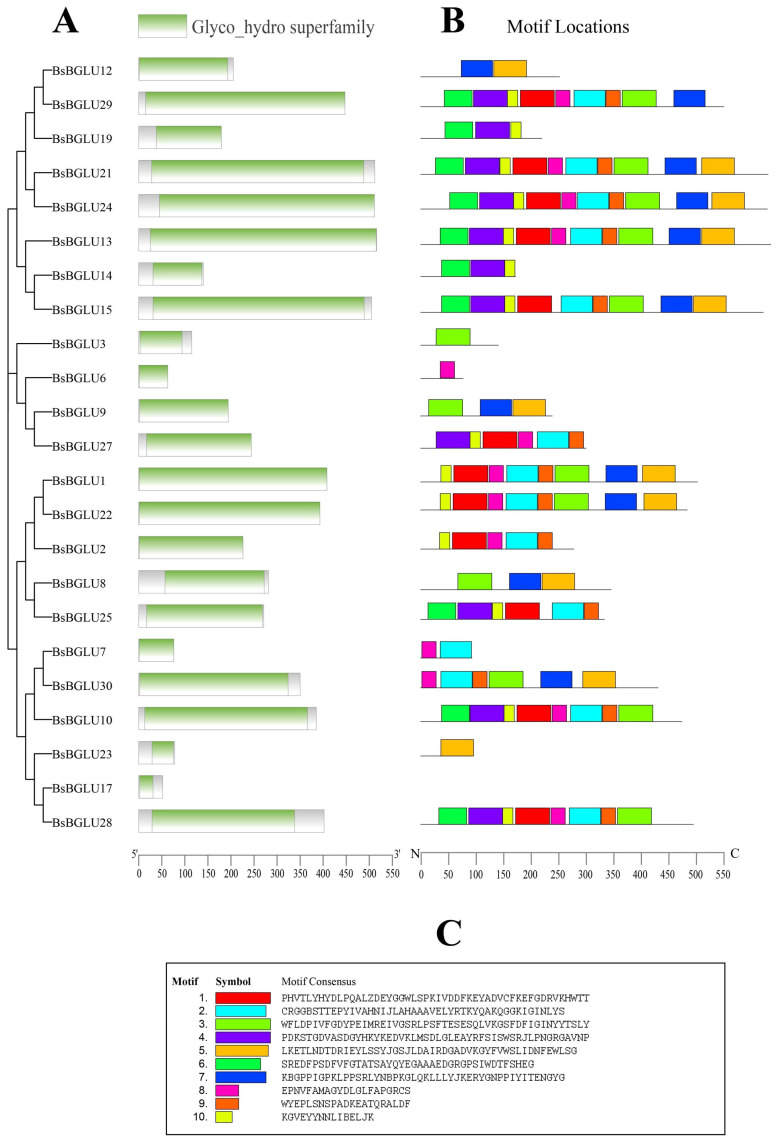
Nucleotide and amino acid sequence characterization of BGLU members. (**A**) The conserved domains of the detected BGLU members. The location (**B**) and sequence (**C**) of the conserved motifs on BGLU protein family. The length and order of the boxes with different colors represent the actual size and location of each motif in protein sequence, respectively.

Subcellular localization predictions indicated diverse locations for BsBGLU proteins, implying distinct functional roles (Figure 1). BsBGLU17 is the shortest protein within the family and lacks a conserved sequence, indicating that it has undergone significant evolutionary changes and may no longer retain its original biological function. BsBGLU13, BsBGLU14, and BsBGLU15 are primarily localized in chloroplasts and extracellular spaces. The presence of a signal peptide (Table S2) indicates that these proteins are transported to chloroplasts or secreted outside the cell following their synthesis. Given their localization in chloroplasts, these proteins are likely involved in processes related to photosynthesis, energy conversion, or metabolism. Their extracellular localization may be associated with functions such as intercellular signaling, material exchange, or defense mechanisms. 

BsBGLU19 is localized in the vacuolar membrane and chloroplasts, and the signal peptide facilitates its entry into these organelles through intracellular transport mechanisms, such as the TOC-TIC complex. In chloroplasts, this protein may participate in photosynthesis, metabolism, or energy conversion, while in vacuoles, it may be involved in material storage, ion balance, or the processing of intracellular waste. BsBGLU21 and BsBGLU24 are found in the vacuolar membrane and extracellular space, and their signal peptides suggest that these proteins are directed to the endoplasmic reticulum (ER) after synthesis, where they undergo processing and modification before being transported to the vacuolar membrane and extracellular space. This represents a typical pathway for secreted proteins, with the signal peptide aiding in the correct localization of nascent polypeptide chains.

The multiple localization of BsBGLU23 indicates that it may perform distinct functions in various cellular compartments. In the nucleus, it may be involved in regulating gene expression or other nuclear processes; in the cytoplasm, it may participate in cellular metabolism or signal transduction; and in the extracellular space, it may play a role in intercellular communication or signaling. This characteristic of multiple localization, combined with the presence of a signal peptide, suggests that BsBGLU23 can respond to different cellular signals or environmental changes, thereby contributing to the cell’s adaptive responses. In mitochondria, the signal peptide may assist the protein BsBGLU27 in entering the mitochondria through specific transport mechanisms, enabling it to fulfill its physiological functions. Collectively, these localizations imply functions related to the secretory pathway and extracellular activities, indicating that these proteins play integral roles in various cellular processes associated with plant growth and development.

#### 2.1.2. Conserved Motif and Gene Structure Analysis of BsBGLU Amino Acid Sequence

**Figure 2:**
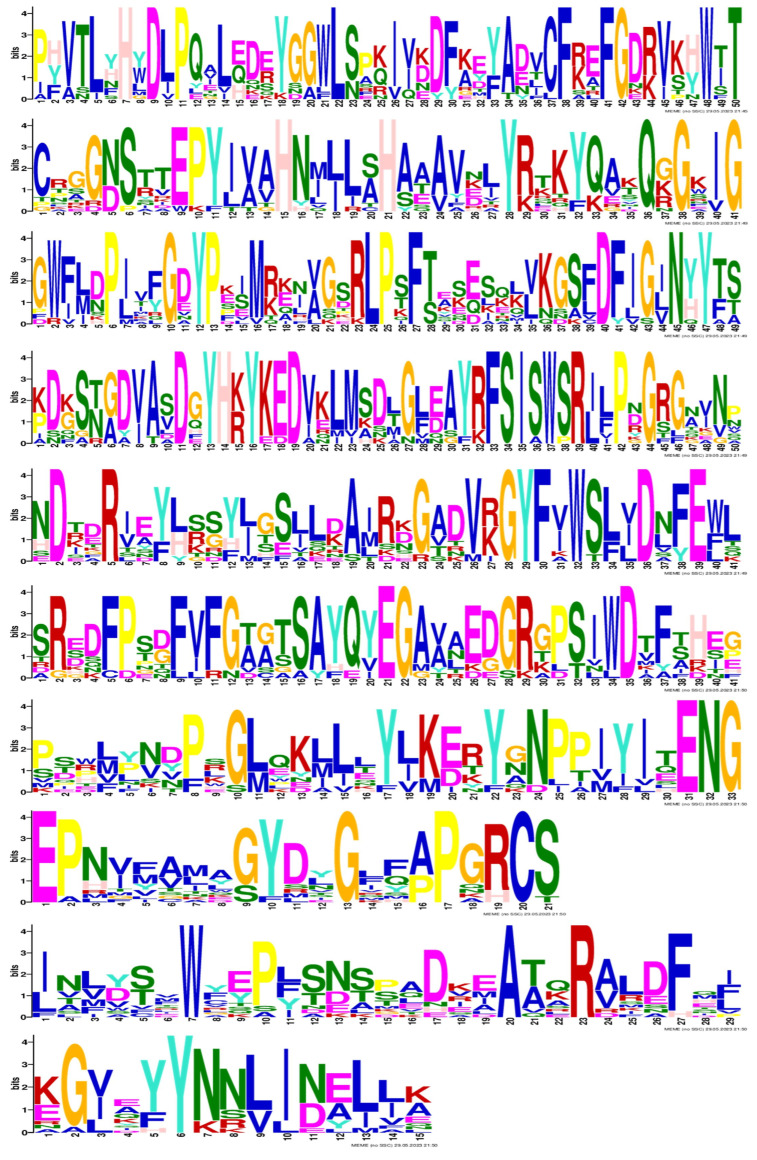
The size of the conserved motifs on BGLU protein family. Motif 1–10 represent different conserved amino acid sequences, and the fewer that amino acid types appear at different positions in each motif, the more conserved the position is.

Conserved motif analysis of BsBGLU proteins was performed using the MEME tool (https://meme-suite.org/meme/tools/meme, accessed on 5 October 2024), identifying 10 distinct motifs (Figure 2). All 23 BsBGLU proteins contained the conserved GH1 domain (PF00232), confirming their classification within the glycoside hydrolase family 1. The identified motifs varied in length from 15 to 50 amino acids, and each BsBGLU protein contained between 2 and 10 motifs. The overall conservation of motif patterns among BsBGLU proteins underscores their potential collective roles in plant physiological processes.

Prominent amino acids E (glutamic acid) and K (lysine) suggest involvement in protein–protein interactions or functional activity in motif 1. Motif 2 shows that Notable S (serine) and T (threonine) indicate potential phosphorylation sites, hinting at a regulatory function. Motif 3 contains G (glycine) and D (aspartic acid), indicating flexibility and potential roles in catalytic functions or ion-binding sites. Basic amino acids R (arginine) and K imply roles in DNA binding or protein–protein interactions in motif 4. Motif 5 shows that hydrophobic amino acids A (alanine) and L (leucine) indicate functions related to protein folding or membrane association. Motif 6 includes the aromatic amino acids F (phenylalanine) and Y (tyrosine), which are likely to play a role in pro-tein–protein interactions or binding sites. N (asparagine) and Q (glutamine) suggest roles in hydrogen bonding and protein–protein interactions in motif 7. Hydrophobic amino acids V (valine) and I (isoleucine) point to functions in protein folding or membrane association in motif 8. Motif 9 has C (cysteine), which is critical for disulfide bond formation, enhancing protein stability, while M (methionine) may have additional roles. Motif 10 shows that H (histidine) is often associated with catalytic and metal-binding sites, while W (tryptophan) may facilitate protein–protein interactions.

BsBGLU13, BsBGLU21, and BsBGLU24 possessed all 10 motifs, indicating a high degree of conservation. Motifs 2 and 10 were the most widely distributed, and the high occurrence of these motifs suggests that they are critical for the structural integrity and catalytic functions of BsBGLU proteins. Interestingly, nine BsBGLU proteins (BsBGLU3, BsBGLU6, BsBGLU7, BsBGLU8, BsBGLU9, BsBGLU12, BsBGLU14, BsBGLU17, and BsBGLU19) contained fewer than four motifs, possibly indicating functional divergence or evolutionary adaptation. The absence of certain motifs in these proteins may reflect gene evolution, resulting in new functions or the loss of original functions due to environmental changes.

#### 2.1.3. Phylogenetic Analysis of BsBGLU Amino Acid Sequence

**Figure 3:**
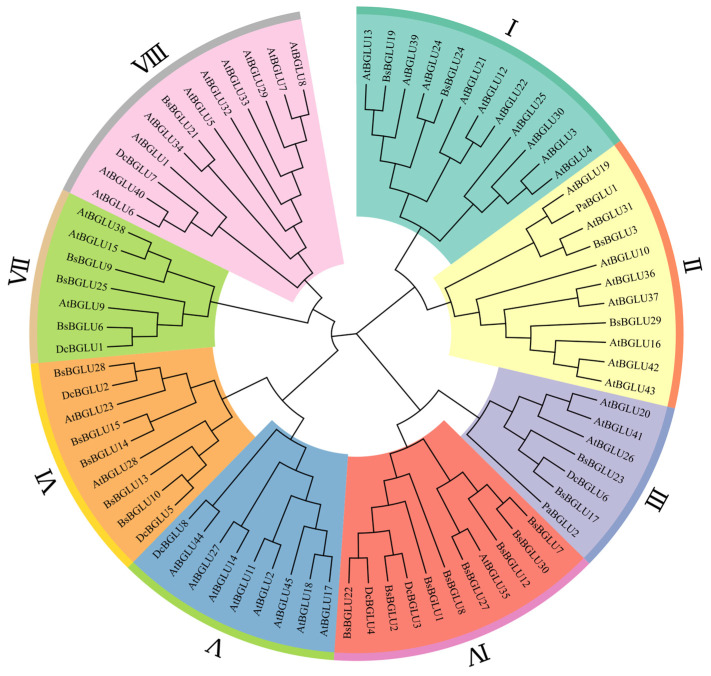
Phylogenetic tree of *BGLU* genes in *B. striata*, *A. thaliana*, *D. catenatum*, and *P. aphrodite*. I–VIII are eight ethnic groups based on evolutionary relationship classification. (The accession number of all sequences used in the phylogenetic analysis in Table S1).

The phylogenetic analysis grouped the 23 BsBGLU proteins into seven distinct clusters (Figure 3). Notably, Cluster V lacked any BsBGLU proteins, suggesting possible gene loss during the evolution of *B. striata* compared to *A. thaliana*. Phylogenetic analysis revealed that BsBGLU proteins within the same clade shared similar motif compositions, supporting the effectiveness of the clustering and suggesting functional similarities among closely related proteins.

In Cluster I, BsBGLU19 showed a high degree of similarity to AtBGLU13, and BsBGLU24 was closely related to AtBGLU24. Similarly, there are similar BsBGLU to AtBGLU and DcBGLU proteins in each cluster. Shorter evolutionary branch lengths indicated closer evolutionary relationships among the proteins. The evolutionary analysis revealed that genes within the same cluster shared similar topological structures and conserved sequences. This suggests that the *BsBGLU* genes and their counterparts in closely related species have shared genetic characteristics and evolutionary paths, reflecting functional conservation within the glycoside hydrolase family 1.

#### 2.1.4. Detection of EST–SSR Polymorphisms in BsBGLU Nucleotide Sequence

Expressed sequence tag–simple sequence repeat (EST–SSR) markers offer advantages such as high polymorphism, diversity, excellent repeatability, and precise, rapid detection. Using the NWISRL website (https://ssr.nwisrl.ars.usda.gov/, accessed on 5 October 2024), SSR loci were identified in 9 out of the 23 *BsBGLU* sequences. Among these, three sequences contained mononucleotide repeats, six sequences had dinucleotide repeats, and one sequence included a trinucleotide repeat. The number of repeat units ranged from 5 to 22.

**Figure 4:**
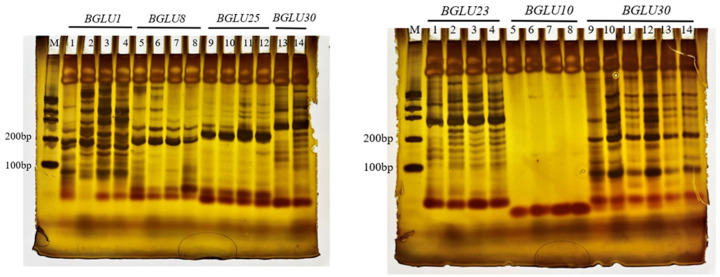
SSR electrophoresis amplification results of four different landraces of *B. striata*. In the left result, loading hole 1–4 are *BsBGLU1* amplified bands in 4 different *B. striata* landraces. The following bands is the same. Loading hole 5–8 are *BsBGLU8* amplified bands in 4 different *B. striata* landraces. Loading hole 9–12 are *BsBGLU25* amplified bands in 4 different *B. striata* landraces. Loading hole 9–12 are *BsBGLU25* amplified bands in 4 different *B. striata* landraces. In the right result, loading hole 1–4 are *BsBGLU23* amplified bands in 4 different *B. striata* landraces. Loading hole 5–8 are *BsBGLU10* amplified bands in 4 different *B. striata* landraces. Loading hole 9–12 are *BsBGLU30* amplified bands in 4 different *B. striata* landraces. Loading hole 13–14 are both *BsBGLU30* repeated results.

Six pairs of SSR primers were designed using Primer3Plus (Table S2) and were consistently amplified across four different B. striata landraces exhibiting distinct phenotypic characteristics. The amplified fragments ranged from 100 to 200 base pairs in length (Figure 4). The SSR frequency, defined as the proportion of Unigenes containing SSRs to the total number of Unigenes, was 39.13%.

The presence of similar *BsBGLU* SSR bands in various landraces suggests that *BsBGLU* genes containing SSR loci are highly conserved. The conservation of SSR fragments indicates strong stability of SSR sequences within biological genomes and adaptability across closely related species. Additionally, the length of the SSR sequences can serve as a criterion for assessing polymorphism. In this study, SSR sizes ranged from 11 to 44 base pairs, and the four *B. striata* landraces exhibited distinct variations. Therefore, SSR primers can serve as effective molecular markers for distinguishing *BsBGLU* family genes in different plant species.

### 2.2. Functional Prediction of Nucleotide and Amino Acid Sequence

#### 2.2.1. Promoter-Based Cis-Element Analysis of *BsBGLU*

**Figure 5:**
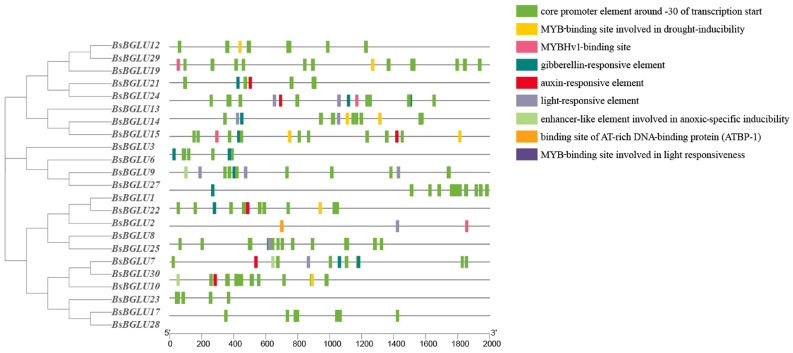
Function of cis-acting elements of *BsBGLU* gene family promoter.

Three major hormone-responsive elements—abscisic acid (ABA), gibberellin (GA), and auxin—were prominently present in the promoter regions (Figure 5). Elements related to anaerobic induction, drought responsiveness, low-temperature responsiveness, defense, and stress were identified. Additionally, a significant number of light-responsive regulatory elements and MYB-binding sites were also present.

Gibberellin-responsive elements were the most prevalent, identified in 13 *BsBGLU* genes. Light-responsive elements were found in eight *BsBGLU* genes. Auxin-responsive elements were present in seven *BsBGLU* genes. BsBGLU10, BsBGLU15, and BsBGLU23 contained enhancer-like elements involved in hypoxia-specific activation. Ten *BsBGLU* genes possessed MYB-binding sites associated with drought induction, and five genes had MYBHv1-binding sites linked to drought and osmotic stress responses [15]. The least common were AT-rich DNA-binding protein (ATBP-1)-binding sites, which play a role in plant gene transcription [16,17].

The presence of plant-hormone-responsive elements suggests that *BsBGLU* genes can respond to fluctuations in hormone levels, thus modulating gene expression. Abscisic acid response elements mediate plant responses to drought and salt stress. MYB-binding sites, along with elements responsive to light, drought, low temperatures, and hypoxia, collectively modulate physiological and morphological changes in plants. The diverse array of cis-acting elements indicates that *BsBGLU* genes play crucial roles in plant growth, development, and stress responses.

#### 2.2.2. Functional Annotation and Protein–Protein Interaction Analysis of BsBGLU

**Figure 6:**
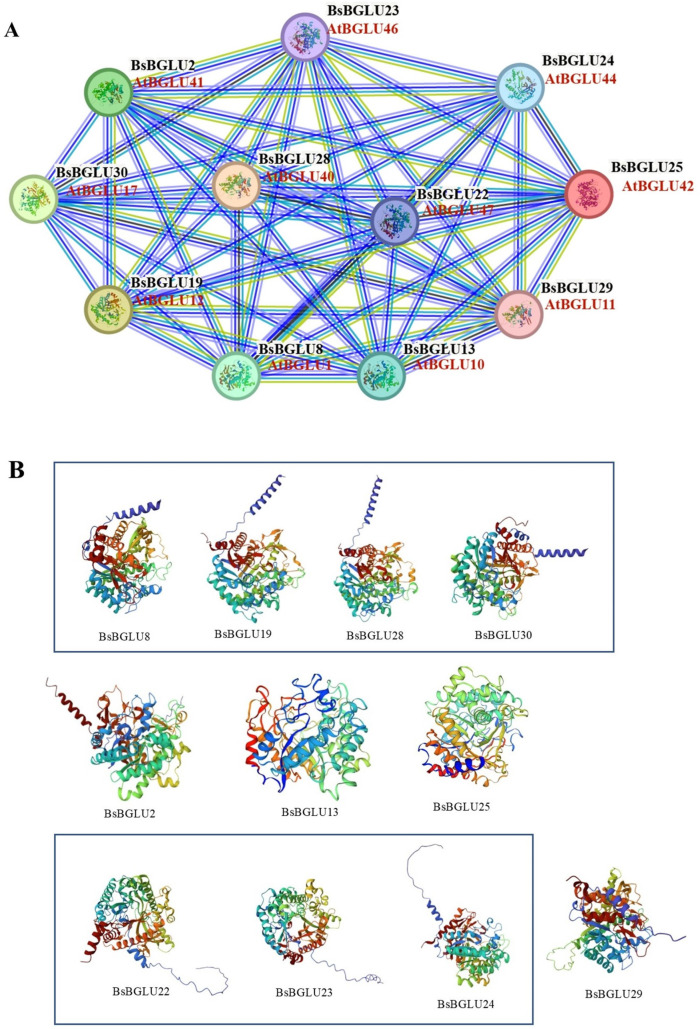
Functional annotation and protein–protein interaction analysis of BsBGLU. (**A**) BsBGLU’s strong interaction protein network with *Arabidopsis* sequence alignment. (**B**) 3D structure prediction of strongly interacting proteins of BsBGLUs.

Gene Ontology (GO) and Kyoto Encyclopedia of Genes and Genomes (KEGG) analyses assigned the 23 BsBGLU proteins to carbohydrate metabolism pathways, specifically phenylpropanoid biosynthesis, starch and sucrose metabolism, and cyanoamino acid metabolism. Protein sequence alignments with *A. thaliana* and *Dendrobium* officinale revealed strong connections among 11 BsBGLU proteins (Figure 6), highlighting their importance in maintaining normal physiological functions in plants.

In the biological process category of GO annotation, 11 proteins were involved in organic compound synthesis, primary metabolic processes, and carbohydrate metabolism. In terms of molecular function, these proteins exhibited catalytic activity related to β-glucosidase activity, O-glycosyl compound hydrolase activity, and glycosyl bond hydrolysis.

Based on the prediction results of interaction network, BsBGLU42 is implicated in the secretion of root-derived phenolics in response to iron ion (Fe) depletion. It also enhances disease resistance against pathogens such as *Botrytis cinerea*, *Hyaloperonospora arabidopsidis*, and *Pseudomonas syringae pv.* tomato DC3000. Furthermore, BsBGLU42 is essential for rhizobacteria-mediated induced systemic resistance (ISR), particularly in the presence of beneficial strains like Pseudomonas fluorescens WCS417r, providing broad-spectrum protection against various pathogens. BsBGLU44 is capable of hydrolyzing a range of substrates, including p-nitrophenyl beta-D-glucoside, p-nitrophenyl beta-D-mannoside, cellobiose, 4-methylumbelliferyl-beta-D-glucoside, laminarin, amygdalin, esculin, and gentiobiose. Similarly, BsBGLU46 hydrolyzes p-nitrophenyl beta-D-glucoside, p-nitrophenyl beta-D-galactoside, and several natural glucosides, such as salicin, p-coumaryl alcohol glucoside, phenyl-beta-D-glucoside, coniferin, syringin, and arbutin. Additionally, these enzymes may play a role in lignification by hydrolyzing monolignol glucosides.

Structural similarities were observed among BsBGLU8, BsBGLU19, BsBGLU28, and BsBGLU30, suggesting that their functions and interactions may be highly similar. Furthermore, all 23 BsBGLU proteins were predicted to have more than eight potential O-linked glycosylation sites. Seventeen BsBGLU proteins possessed at least one N-linked glycosylation site (Figure S1). This supports the notion that many BsBGLUs may utilize secretory pathways to hydrolyze their substrates [9,18].

These comprehensive findings suggest that BsBGLUs collectively modulate the biosynthesis of secondary metabolites and other metabolic pathways in plants, facilitating carbohydrate conversion and contributing to plant growth and development.

### 2.3. BsBGLU Gene Function Analysis and Verification Result

#### 2.3.1. Double Analysis and of the *BsBGLU* Gene Family Expression

**Figure 7:**
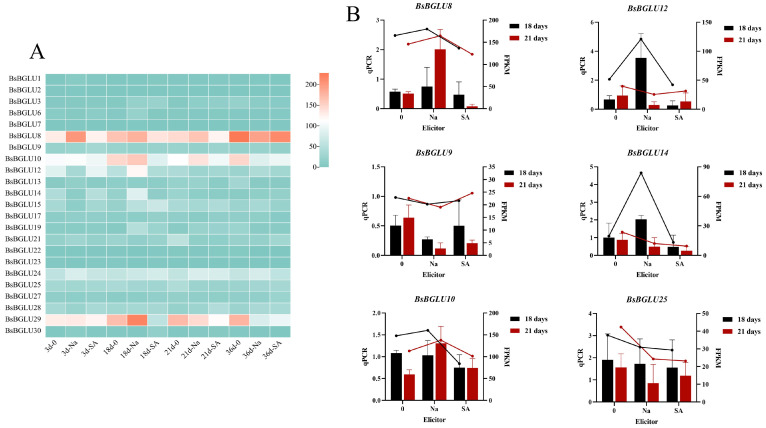
Double analysis of the *BsBGLU* gene family expression. (**A**) Expression patterns of *BsBGLU* gene family members in different stages of suspension culture. The abscissa represents different suspension culture days and culture conditions, and each three columns is a group. (**B**) The left ordinate represents the FPKM value of transcriptome sequencing, the right ordinate corresponds to the RT-qPCR expression, and the abscissa represents the treatment conditions of different elicitors. Black and red in (**B**) corresponds to 18 and 21 days in (**A**).

We analyzed the expression profiles of 23 *BsBGLU* genes under three distinct culture conditions—control, sodium acetate (NaAc) treatment, and salicylic acid (SA) treatment—across four developmental stages of *B. striata* suspension culture. Utilizing Fragments Per Kilobase of transcript per Million mapped reads (FPKM) values for quantification (Figure 7A), we aimed to determine how these genes respond to different elicitors and developmental cues.

Our results revealed that treatment with NaAc significantly upmodulated the expression of *BsBGLU8*, *BsBGLU10*, *BsBGLU12*, *BsBGLU24*, and *BsBGLU29* compared to the control group. This suggests that NaAc acts as an effective elicitor, enhancing the expression of specific *BsBGLU* genes associated with secondary metabolite biosynthesis. Conversely, during SA treatment, these same genes exhibited decreased expression, indicating a differential regulatory mechanism in response to SA. This differential expression underscores the sensitivity of *BsBGLU* genes to specific chemical stimuli, which in turn influences the growth of suspension cells and the production of valuable secondary metabolites.

Over the course of the suspension culture, the majority of *BsBGLU* genes demonstrated an upmodulated expression pattern. This general uptrend implies that these genes may play a direct or indirect role in enhancing secondary metabolite production as the culture progresses. Notably, *BsBGLU8*, *BsBGLU10*, and *BsBGLU29* showed elevated expression levels at various developmental stages, highlighting their potential significance throughout the suspension culture process of *B. striata.* The consistent high expression of these genes suggests they could be key modulators in the metabolic pathways leading to secondary metabolite accumulation.

To validate the reliability of our transcriptome sequencing data and confirm the observed expression patterns, we selected six genes at random for quantitative real-time PCR (qRT-PCR) analysis, using the *18S rRNA* gene as an internal reference control. The qRT-PCR results aligned closely with the transcriptome data, reaffirming the expression trends of the differentially expressed genes (DEGs) (Figure 7B). Statistical correlation analysis using SPSS 29.0 (IBM Corp., Armonk, NY, USA, Version 29.0) software revealed a strong positive relationship between qRT-PCR and FPKM values. Specifically, for the 18-day culture period, the correlation coefficient was 0.884 (*p* < 0.01), indicating a highly significant correlation. For the 21-day period, the correlation coefficient was 0.685 (*p* < 0.05), also reflecting a significant positive correlation. These findings confirm the consistent expression of the *BsBGLU* gene family and validate the dependability of our transcriptome data.

#### 2.3.2. Correlation Analysis Between BsBGLU Gene Expression and Metabolite Accumulation

**Figure 8:**
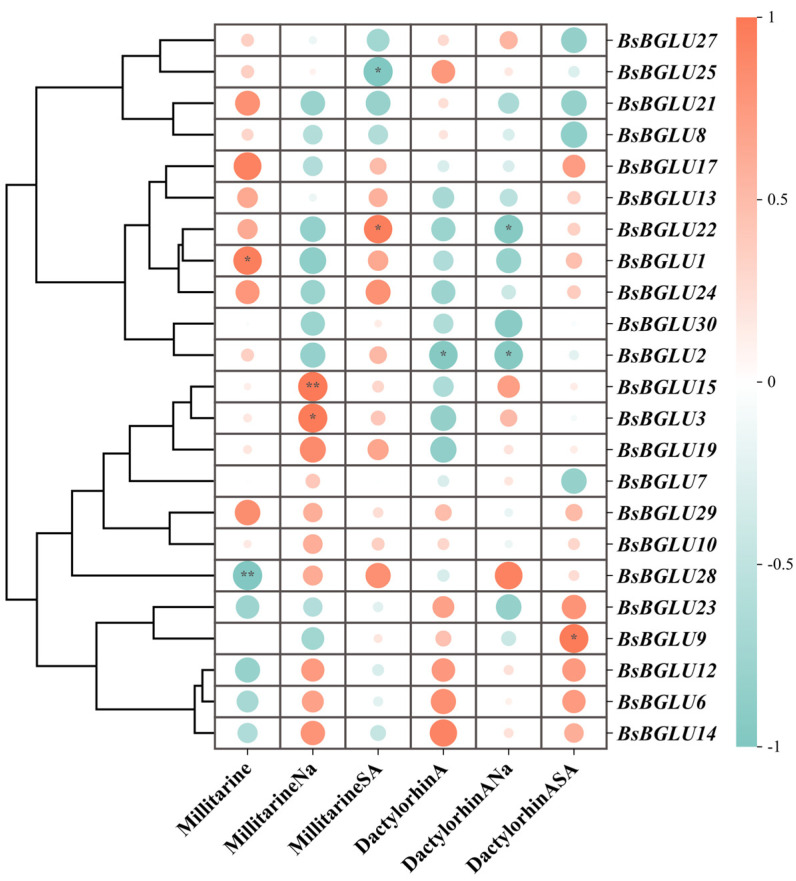
The correlation heatmap between the expression of *BsBGLU* gene and the synthesis of militarine and dactylorhin A under three culture modes. Militarine and dactylorhin A were used as blank controls, Na was induced by 150 mmol/L NaAC, and SA was induced by 15 mmol/L salicylic acid. “*” means significant at the 0.05 level (*p* < 0.05), “**” means significant at the 0.01 level (*p* < 0.01).

We analyzed the correlation between the FPKM values of 23 *BsBGLU* genes and the contents of militarine and dactylorhin A in *B. striata* subjected to SA and NaAc treatments. A heatmap was generated to visualize the expression patterns and metabolite accumulation (Figure 8). Our findings indicated that all 23 *BsBGLU* genes responded to the elicitor treatments, exhibiting varied associations with the accumulation of militarine and dactylorhin A. In the control group without elicitor treatment, genes such as *BsBGLU3*, *BsBGLU15*, and *BsBGLU19* showed a weak positive correlation with militarine accumulation and a negative correlation with dactylorhin A. Following NaAc treatment, the expression levels of these genes were notably increased. This upmodulation was positively associated with the production of both militarine and dactylorhin A. Specifically, *BsBGLU15* demonstrated a strong positive correlation with militarine synthesis (r = 0.995 **, *p* < 0.01), suggesting its significant involvement in metabolite biosynthesis under NaAc elicitation.

Similarly, genes *BsBGLU3*, *BsBGLU6*, *BsBGLU12*, *BsBGLU14*, and *BsBGLU28* exhibited negative correlations with militarine in the control group. After NaAc treatment, these genes showed positive correlations with militarine accumulation, indicating their responsiveness to NaAc and potential roles in stimulating militarine synthesis either directly or indirectly. Notably, *BsBGLU28* displayed a negative association with the production of militarine and dactylorhin A in the control group (r = −0.992**, *p* < 0.01). Upon NaAc and SA treatments, *BsBGLU28* exhibited a positive regulatory effect, suggesting its ability to respond to changes in NaAc and SA concentrations. This response may facilitate the synthesis of militarine, indicating that *BsBGLU28* could be a key metabolic regulatory gene adapting to adverse environmental conditions.

Conversely, genes *BsBGLU8*, *BsBGLU21*, *BsBGLU25*, and *BsBGLU27* showed negative correlations with the synthesis of militarine and dactylorhin A following SA treatment. This observation suggests that these genes may indirectly impede the production of these metabolites, possibly reallocating resources to support the growth and development of *B. striata* under favorable conditions.

#### 2.3.3. Analysis of Key BsBGLU Gene Expression in Different Landraces

**Figure 9:**
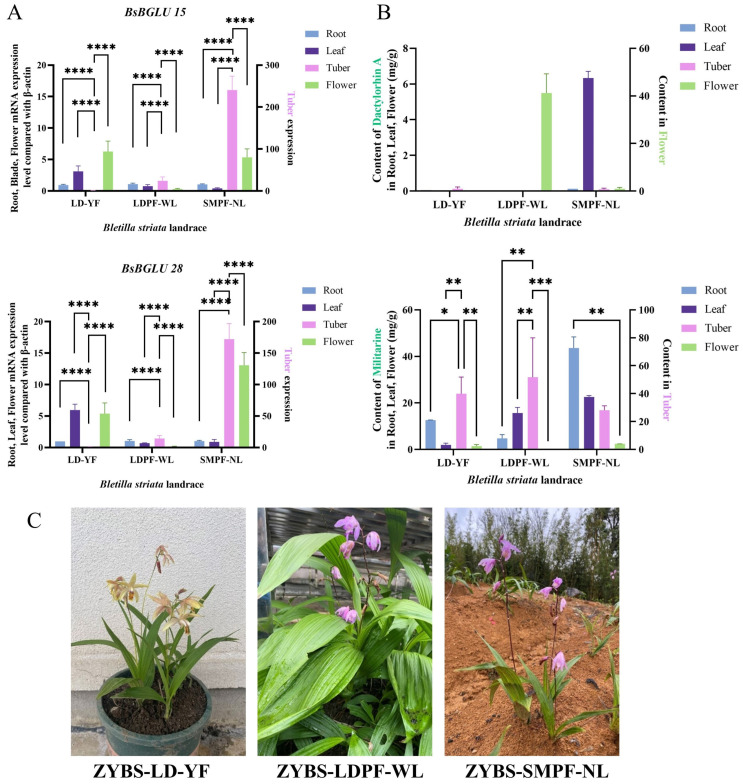
Key gene expression profile and metabolite accumulation of *B. striata.* (**A**) The expression of *BsBGLU15* and *BsBGLU28* genes in four different tissues of three different landraces. (**B**) Accumulation for pharmaceutical metabolites in four different tissues of three different landraces. (**C**) Three kinds of *B. striata* landrace. ZYBS-LD-YF refers to yellow flower *B. striata*, ZYBS-LDPF-WL refers to purple flower *B. striata* with wide leaves, and ZYBS-SMPF-NL refers to purple flower *B. striata* with narrow leaves. “*” means significant statistical differences between the groups at the 0.05 level (*p* < 0.05), “**” means significant at the 0.01 level (*p* < 0.01), “***” means significant at the 0.001 level (*p* < 0.001), “****” means significant at the 0.0001 level (*p* < 0.0001). ZYBS-LD-YF in (**C**) correspond to LD-YF in (**A**,**B**), the following is the same. ZYBS-LDPF-WL in (**C**) correspond to LDPF-WL in (**A**,**B**), ZYBS-SMPF-NL in (**C**) correspond to SMPF-NL in (**A**,**B**).

To further elucidate the roles of *BsBGLU15* and *BsBGLU28*, which exhibited strong correlations with metabolite accumulation (*p* < 0.01) under elicitor treatments, we analyzed their expression profiles across four different tissues—roots, tubers, leaves, and flowers—from three *B. striata* germplasms (ZYBS-LD-YF, ZYBS-LDPF-WL, and ZYBS-SMPF-NL) (Figure 9A). *BsBGLU15* and *BsBGLU28* showed distinct and tissue-specific expression patterns. The expression levels of these genes in tubers were ranked as follows: ZYBS-SMPF-NL > ZYBS-LDPF-WL > ZYBS-LD-YF. This pattern suggests that they may play crucial roles in controlling the accumulation of therapeutic active components, particularly in germplasms with purple flowers known for enhanced medicinal properties. It is plausible that *BsBGLU15* and *BsBGLU28* are specialized metabolic regulatory genes.

Furthermore, the expression levels of *BsBGLU15* and *BsBGLU28* in flowers were higher than those in roots and leaves, indicating their potential involvement in pigmentation and aroma production. Consequently, they likely contribute to the modulation of floral color and scent. These results demonstrate that the expression patterns of *BsBGLU* genes are diverse and tissue-specific. Notably, the similar expression patterns of *BsBGLU15* and *BsBGLU28* suggest that they play critical roles in plant polysaccharide metabolism and morphological development.

#### 2.3.4. The Relationship Between *BsBGLU* Genes and Accumulation

The accumulation of militarine and dactylorhin A in various tissues was assessed using mass spectrometry (Figure 9B). Militarine was distributed in all parts except the flowers of ZYBS-LDPF-WL, whereas dactylorhin A was only detected in the flowers of ZYBS-LDPF-WL and the leaves of ZYBS-SMPF-NL. Both dactylorhin A and gastrodin are precursors for militarine synthesis [2,4]. Gastrodin was not detected in any germplasms or tissues, possibly due to its low concentration or rapid conversion to other metabolites.

In the flowers of ZYBS-LDPF-WL, the accumulation of militarine was 0 mg/g, while dactylorhin A reached 6.34 mg/g, suggesting it is in the process of synthesizing militarine pathway. The tubers exhibited significantly higher militarine accumulation compared to other tissues. Additionally, militarine levels in the leaves of ZYBS-LDPF-WL and ZYBS-SMPF-NL were higher than those in ZYBS-LD-YF, whereas in roots, ZYBS-LD-YF had higher levels than the other two germplasms. These findings indicate that militarine not only accumulates in tubers but is also synthesized in other parts of *B. striata*.

Notably, the leaves of ZYBS-SMPF-NL showed significantly higher accumulation of militarine and dactylorhin A compared to the other germplasms, while its tubers had considerably lower militarine levels. Combined with the high expression of *BsBGLU15* and *BsBGLU28* in the tubers of ZYBS-SMPF-NL and minimal expression in the leaves, it is speculated that these genes may play a negative regulatory role in militarine synthesis. This hypothesis is further supported by gene expression and metabolite accumulation patterns in the other germplasms. For instance, *BsBGLU15* and *BsBGLU28* were expressed at low levels in the tubers of ZYBS-LDPF-WL and ZYBS-LD-YF, where militarine accumulated highly. Conversely, they were expressed in the leaves and flowers of ZYBS-LD-YF, where militarine accumulation was low. These observations suggest a negative correlation between the expression of *BsBGLU15* and *BsBGLU28* and militarine levels, indicating that they may directly or indirectly act as negative modulators in the synthesis of militarine.

The results of gene expression analysis following elicitor treatment (Figure 8) indicate that NaAc influences the expression of *BsBGLU15* and *BsBGLU28*. Specifically, treatment with 150 mmol/L NaAc demonstrated a significant positive correlation between the expression levels of *BsBGLU15* and *BsBGLU28* and the accumulation of militarine and dactylorhin A. This suggests that the elicitor NaAc can regulate metabolite synthesis by modulating the expression of *BsBGLUs*. For instance, 150 mmol/L NaAc appears to inhibit the expression of *BsBGLU15* and *BsBGLU28* while simultaneously promoting the accumulation of militarine and dactylorhin A.

## 3. Discussion

### 3.1. Systematic Genome-Wide Analysis of the BGLU Gene Family

The biosynthesis of pharmaceutical metabolites in medicinal plants is a complex process that involves a myriad of enzymes and regulatory pathways. Among these plants, *Bletilla striata* has garnered significant attention due to its extensive medicinal efficacy and potential for clinical applications. This valuable Chinese herbal medicine is known for producing several important pharmaceutical metabolites, including polysaccharides, dactylorhin A, militarine, and gastrodin [19,20,21], which contribute to its therapeutic properties.

**Figure 10:**
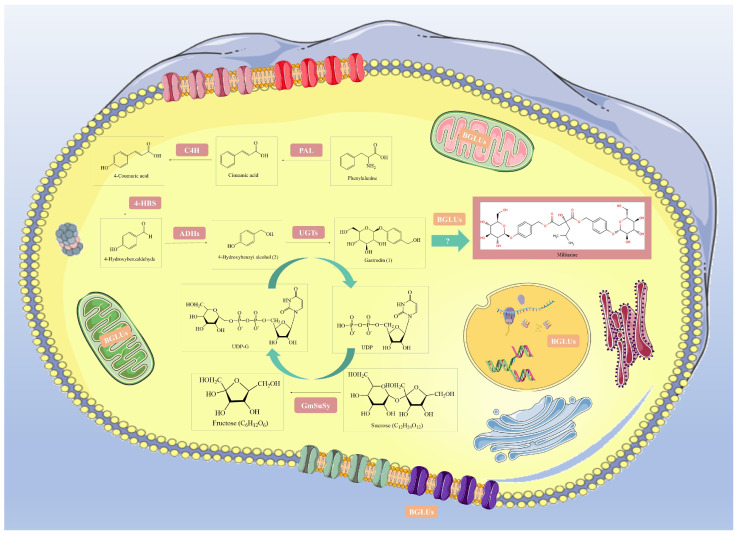
The cytoplasmic matrix is responsible for the metabolic production of phenylpropane polysaccharides and militarine.

Previous studies have primarily focused on transcriptome analyses of *B. striata* to identify potential genes and pathways involved in the production of these metabolites [1,2]. While these studies have provided insights into the biosynthetic processes at the transcriptomic level, a comprehensive understanding of the gene families involved at the genomic level remains limited. Specifically, the β-glucosidase (BGLU) gene family, known for its versatile roles in plant growth, secondary metabolite production, and responses to biotic and abiotic stressors (Figure 10) [22,23], has not been thoroughly examined in *B. striata.*

The advent of advanced whole-genome sequencing technologies has opened new avenues for the systematic analysis of gene families across various plant species [23,24,25,26,27]. Notably, orchids such as *Apostasia shenzhenica*, *Vanilla planifolia*, *Phalaenopsis equestris*, *Phalaenopsis aphrodite*, and *Dendrobium catenatum* have been found to possess a significantly lower number of *CH1-BGLU* genes compared to other plants, suggesting gene loss or pseudogenization during evolution [28,29,30,31]. This underscores the importance of investigating the *BGLU* gene family within orchids to understand their evolutionary adaptations and functional diversification.

In this context, our study aimed to fill this research gap by conducting a systematic analysis of the *BGLU* gene family in B. striata. We identified a total of 23 *BsBGLU* genes through multiple sequence alignment and phylogenetic analysis. These genes exhibited significant sequence similarity and conserved domains, indicating their evolutionary conservation within the species. Subcellular localization predictions revealed that BsBGLU proteins are primarily localized in the cytoplasm, chloroplasts, and vacuoles, where they likely participate in catalytic reactions essential for metabolic processes.

Further analysis showed that all 23 BsBGLU proteins are involved in the carbohydrate metabolic pathway, with 11 of them exhibiting distinct hydrolase activity. Notably, all BsBGLU proteins possess more than eight predicted O-glycosylation sites, and most contain at least one predicted N-glycosylation site. This supports the hypothesis that several β-glucosidase-like enzymes in *B. striata* may break down substrates via secretory pathways, contributing to the plant’s metabolic versatility.

By examining their evolutionary relationships, protein structural functions, and expression patterns across various tissues and in response to biotic stressors, we have provided a comprehensive overview of the *BsBGLU* gene family. Additionally, correlation analysis between gene expression and metabolite levels led to the discovery of two potential genes, *BsBGLU15* and *BsBGLU28*, implicated in the biosynthesis of active metabolites. The elicitor NaAc can regulate metabolite synthesis by modulating the expression of *BsBGLUs*. This systematic analysis not only enhances our understanding of the biological roles of the *BsBGLU* gene family but also identifies possible targets for molecular breeding aimed at improving the production of valuable pharmaceutical compounds.

### 3.2. Role of BsBGLUs in Plant Metabolite Accumulation in Response to Stress

The accumulation of plant metabolites is a dynamic process influenced by various environmental factors and stress conditions [32]. Introducing elicitors into plant cell cultures has been a proven strategy to enhance the production of valuable secondary metabolites [33,34]. Elicitors such as SA and NaAc can stimulate plant defense responses, leading to the altered expression of genes involved in metabolite biosynthesis [35,36,37,38].

In this study, we investigated the role of *BsBGLU* genes in the accumulation of pharmaceutical metabolites in *B. striata* under stress conditions induced by elicitor treatments. By analyzing suspension cells cultured under three different conditions, we observed that the 23 identified *BsBGLU* genes could be classified into seven major clusters based on their amino acid sequence similarities and motif compositions. This classification reflects a rich diversity that mirrors the evolutionary adaptations of *B. striata*.

Our investigation into the conserved domains, gene structures, genomic localization, cis-elements, expression profiles, and the modulation of the metabolite accumulation of *BsBGLUs* revealed intriguing insights into their functional roles. Notably, certain *BsBGLU* genes possess cis-acting elements in their promoters that are responsive to plant hormones such as auxin and gibberellin, as well as environmental stress factors. This indicates a complex regulatory network where *BsBGLUs* play critical roles in plant physiology and adaptability.

Through the integration of existing RNA-seq data and qPCR validation, we demonstrated that the expression levels of *BsBGLU* genes are significantly affected by SA and NaAc treatments [2]. Specifically, treatment with 150 mmol/L NaAc demonstrated a significant positive correlation between the expression levels of *BsBGLU15* and *BsBGLU28* and the accumulation of militarine and dactylorhin A; *BsBGLU15* and *BsBGLU28* emerged as key genes with negative regulatory roles in the biosynthesis of militarine and dactylorhin A. The inverse correlation between the expression of these genes and the accumulation of metabolites suggests that they may act as bottlenecks in the metabolic pathways, potentially redirecting resources in response to stress conditions.

While our findings indicate significant sequence similarities between BsBGLUs and BGLUs in other plant species, the observed functional diversity—particularly in tissue-specific and stress-responsive expressions—highlights the unique roles of BsBGLUs in *B. striata.* This functional divergence calls for a deeper exploration of their mechanisms of action, which could unveil novel targets for genetic manipulation.

Future research should aim to elucidate the precise pathways through which *BsBGLU15* and *BsBGLU28* influence metabolite biosynthesis and stress responses. In addition, further exploration and verification of the function and mechanism of other BsBGLUs are required. Understanding these mechanisms could pave the way for biotechnological innovations, such as developing *B. striata* varieties with the enhanced production of pharmaceutical compounds or improved stress resilience.

## 4. Materials and Methods

### 4.1. Analytical Method of Nucleotide and Amino Acid Sequence Characteristics

#### 4.1.1. Plant Material and Growth Conditions

*Bletilla striata* capsules were collected from the germplasm resource garden at Zunyi Medical University, Xinpu District, Zunyi City, Guizhou Province, China (latitude 27°42′ N, longitude 107°01′ E). Seeds were cultured in suspension for 45 days following established protocols [13]. During the second subculture phase, from day 0 post-inoculation (0 dpi) to 45 dpi, cultures were treated with either 150 μmol/L sodium acetate (NaAc) or 15 μmol/L salicylic acid (SA). Samples were randomly collected at 3 dpi, 18 dpi, 21 dpi, and 36 dpi, with three biological replicates at each time point. Total RNA was extracted from each sample using liquid nitrogen grinding and pooled in equal amounts for high-throughput sequencing. Sequencing was performed on the Illumina X Plus platform, and de novo assembly of the transcriptome was conducted using Trinity software (trinityrnaseq_r2013-02-25 version), resulting in the *B. striata* transcriptome dataset [14]. The assembled data were used for exploring on *BsBGLU* genes.

#### 4.1.2. Genome-Wide Identification of *BsBGLUs*

To identify potential *β-glucosidase (BGLU)* genes of the glycoside hydrolase family 1 (GH1) in *B. striata*, we downloaded the hidden Markov model (HMM) profile of Glyco_hydro_1 (PF00232) from the Pfam database (http://www.ebi.ac.uk/interpro/entry/pfam/#table, accessed on 6 July 2023). The HMMER software suite v3.2.1 (http://hmmer.org/download.html, accessed on 6 July 2023) was employed to search for BGLU candidates. The candidate objects were verified through alignment using NCBI BLAST (https://novopro.cn/blast/, accessed on 5 October 2024). Open reading frames (ORFs) of all candidate BsBGLU sequences were predicted using the ORF Finder tool (https://www.ncbi.nlm.nih.gov/orffinder/, accessed on 7 July 2023). Physical and chemical properties, including molecular weight, amino acid length, isoelectric point, and grand average of hydropathicity (GRAVY), were analyzed using the ExPASy ProtParam tool (https://web.expasy.org/protparam/) [39]. Secondary structure predictions were performed using SOPMA (https://npsa-prabi.ibcp.fr/cgi-bin/npsa_automat.pl?page=npsa_sopma.html, accessed on 5 October 2024) [40], and subcellular localization was predicted with WoLF PSORT (https://wolfpsort.hgc.jp/, accessed on 5 October 2024) [41].

#### 4.1.3. Gene Structure and Motif Analysis of BsBGLUs

Conserved domains within the BsBGLU proteins were identified using the NCBI Conserved Domain Search tool (https://ncbiinsights.ncbi.nlm.nih.gov/tag/conserved-domains-database-cdd/, accessed on 5 October 2024). Motif analysis was conducted using the MEME Suite [42], setting the maximum number of motifs to 10. Signal peptides and transmembrane domains were predicted using SignalP 6.0 and TMMOD [43,44], respectively.

#### 4.1.4. Multiple Sequence Alignment and Phylogenetic Analysis

To investigate the classification and phylogenetic relationships of BGLU peptides in *B. striata* and other species, the BGLU protein sequences from the model of *Arabidopsis thaliana* were obtained from the TAIR database (http://www.arabidopsis.org/, accessed on 6 August 2023), while sequences from *Phalaenopsis aphrodite* and *Dendrobium catenatum*, two species also from Orchidaceae, were retrieved from NCBI. Multiple sequence alignments were performed using Clustal Omega (http://clustal.org/omega/, accessed on 5 October 2024), and a phylogenetic tree was constructed using MEGA-X software [45] with the neighbor-joining method and 1000 bootstrap replicates. The tree was enhanced for visualization using the tvBOT online tool (https://www.chiplot.online/, accessed on 5 October 2024) [46].

#### 4.1.5. Detection and Validation of EST–SSR Markers

Simple sequence repeat (SSR) loci within the 23 *BsBGLU* sequences were identified using MISA (https://webblast.ipk-gatersleben.de/misa/, accessed on 5 October 2024) with default parameters [47]. Specific primers for the SSR loci were designed using Primer3Plus (https://www.primer3plus.com/, accessed on 5 October 2024) [48] (Table S3). To verify the conservation and polymorphism of these SSR markers, leaf samples from four different *B. striata* landraces were collected from our germplasm garden. PCR amplification was performed, and the products were analyzed by polyacrylamide gel electrophoresis (PAGE).

### 4.2. Functional Prediction Based on Sequence Characteristics

#### 4.2.1. Analytical Method of Promoter-Based Cis-Element in BsBGLU

Cis-acting elements within the 2000 bp upstream promoter regions of the *BsBGLU* genes were analyzed using PlantCARE (http://bioinformatics.psb.ugent.be/webtools/plantcare/html/, accessed on 5 October 2024) [49], and visualization was performed with TBtools-II v2.056 [50].

#### 4.2.2. Analytical Method of Functional Annotation and Protein–Protein Interaction in BsBGLU

Gene Ontology (GO) classification and Kyoto Encyclopedia of Genes and Genomes (KEGG) enrichment analyses of the BsBGLU peptides were conducted using the STRING 12.0 database (https://cn.string-db.org/, accessed on 5 October 2024) [51] with default parameters, selecting *A. thaliana* as the reference. The DAVID tool (Database for Annotation, Visualization, and Integrated Discovery) was utilized for functional annotation and pathway prediction (https://davidbioinformatics.nih.gov/, accessed on 5 October 2024) [52]. Predicted pathways were generated by comparing differentially expressed cytoplasmic and nuclear proteins with the KEGG pathway database. Protein–protein interaction (PPI) networks were constructed using STRING 12.0. N-linked glycosylation sites were predicted using NetNGlyc 1.0, and O-linked glycosylation sites were detected using the YinOYang 1.2 server (https://services.healthtech.dtu.dk, accessed on 6 February 2024).

### 4.3. BsBGLU Gene Function Analysis and Verification

#### 4.3.1. Detection of Pharmaceutical Metabolite Accumulation by HPLC

Metabolites from suspension-cultured cells were collected at 3, 18, 21, and 36 dpi, with three biological replicates at each time point, and quantified using a Waters e2695 high-performance liquid chromatography (HPLC) system. The standards (dactylorhin A and militarine) were accurately weighed and, respectively, dissolved in methanol to obtain the single high-concentration standard solutions. Both standard solutions had a concentration of 0.5 mg/mL and were stored in accordance with the instructions of manufacturer. The separations of two components were achieved on a BEH C18 column ACQUITY UPLC^®^ (2.1 × 100 mm, 1.7 µm manufactured by Waters Corporation in Milford, Massachusetts, United States). The column temperature was 30 °C, and the injection volume was 5.00 μL. The mobile phases were delivered at a flow rate of 0.3 mL/min, and those consisted of 0.1% formic acid water (A) and acetonitrile (B) with the gradients: 0–10 min, 20% A, 80% B; 10–25 min, 50% A, 50% B; 27–30 min, 95% A, 5% B; 32–45 min, 20% A, 80% B.

#### 4.3.2. Detection of Pharmaceutical Metabolite Accumulation by LC–MS

Root, tuber, leaf, and flower tissues from three distinct *B. striata* landraces were collected for metabolite quantification using an Agilent 6545 Q-TOF liquid chromatography–mass spectrometry (LC–MS) system. Analytes were quantified by the multiple reaction monitoring (MRM) mode in both positive and negative ion detection modes. The three standards were accurately weighed and, respectively, dissolved in methanol to obtain the single high-concentration standard solutions (dactylorhin A 0.5 mg/mL, gastrodin 0.4 mg/mL, and militarine 0.25 mg/mL). The following settings were used: Agilent Eclipse Plus C18 (2.1 × 100 mm, 1.8 μm); flow rate: 0.5 mL/min; mobile phase: water (0.1% formic acid) (A), 100% acetonitrile (B); column temperature 30 °C; wavelength: 220 nm, 254 nm; the injection volume of the test sample was 5.00 uL, with the gradients: 0–0.5 min 90% A, 10% B; 0.5–6 min 100% B; 6–8 min, 90% A, 10% B.

#### 4.3.3. *BsBGLU* Gene Expression Profile Analysis in Different Tissues

To explore the functions of *BsBGLUs*, we analyzed their expression profiles in *B. striata* suspension-cultured cells at four time points: 3 dpi, 18 dpi, 21 dpi, and 36 dpi. Heat maps were generated using tvBOT [46]. Six genes were randomly selected for detecting their expression profiles via quantitative real-time PCR (qRT-PCR). Specific primers for qRT-PCR were designed using NCBI Primer-BLAST (https://ncbiinsights.ncbi.nlm.nih.gov/2017/06/28/e-pcr-is-retiring-use-primer-blast/, accessed on 5 October 2024) (Table S2).

#### 4.3.4. Analysis of Correlation Between BsBGLU Expression and Metabolite Accumulation

The metabolite accumulation of suspension-cultured cells at four time points was calculated by HPLC test results. The correlation between *BsBGLU* gene expression levels FPKM (Fragments Per Kilobase of transcript per Million mapped reads) and the content of medicinal metabolites, dactylorhin A and militarine, under elicitor treatments (150 μmol/L NaAc and 15 μmol/L SA) was analyzed using SPSS 29.0 (IBM Corp., Armonk, NY, USA) and tvBOT visualized by correlation heat map [46].

#### 4.3.5. Analysis of Relationship Between Key *BsBGLUs* and Accumulation

To assess the accumulation of dactylorhin A, militarine, and gastrodin and their association with *BsBGLU* gene expression in four different tissues, we analyzed three distinct *B. striata* landraces. Based on the results of the correlation heat map analysis, we selected the BsBGLU genes that are strongly correlated with modulation. Their expression profiles in four tissues of *B.striata* from three landraces were determined using quantitative real-time PCR (qRT-PCR). Further, the metabolite accumulation of different landraces of tissues was calculated by LC–MS test results, and the potential role in the biosynthesis of secondary metabolites was elucidated by comprehensively analyzing the relationship between the expression of the selected *BsBGLU* genes and the metabolite accumulation. 

## 5. Conclusions

This study presents a comprehensive analysis of the *BsBGLU* gene family in *B. striata*, shedding light on their evolutionary relationships, structural characteristics, and functional roles in secondary metabolism and stress responses. By identifying and characterizing 23 *BsBGLU* genes, we have enriched the understanding of how these genes contribute to the biosynthesis of valuable pharmaceutical metabolites.

We report the discovery of *BsBGLU15* and *BsBGLU28* as potential negative modulators of metabolite accumulation. Additionally, the elicitor NaAc can regulate metabolite synthesis by modulating the expression of *BsBGLUs*, which offers new perspectives on the metabolic modulation within *B. striata*. These findings hold significant implications for both ecological studies and commercial applications. From an ecological standpoint, understanding how *B. striata* responds to environmental stressors at the molecular level enhances our knowledge of plant adaptability and survival strategies. Commercially, these insights provide a foundation for molecular breeding programs aimed at increasing the yield of medicinal compounds, thereby enhancing the economic value of *B. striata.*

In conclusion, our research not only fills a critical gap in the genomic understanding of *B. striata* but also positions the species as a promising model for biotechnological advancements in plant-based therapeutics. The potential applications of the *BsBGLU* gene family in enhancing stress tolerance and optimizing metabolite production underscore their broader significance. This makes them valuable targets for future research aimed at harnessing the medicinal potential of this remarkable orchid.

## Figures and Tables

**Table 1:** Physical and chemical properties of BGLU protein.

Protein Name	AA Number	MW (Da)	PI	GRAVY	Instability Index	Alpha Helix	Extended Strand	Beta Turn
BsBGLU1	408	46,641.26	9.11	−0.217	22.92	35.54%	17.65%	6.62%
BsBGLU2	226	26,004.44	5.83	−0.376	34.02	40.27%	16.81%	6.19%
BsBGLU3	115	13,091.96	9.52	−0.470	30.11	31.30%	14.78%	6.09%
BsBGLU6	63	7033.89	4.77	−0.256	30.66	23.81%	22.22%	11.11%
BsBGLU7	76	8504.68	7.79	−0.179	36.32	26.32%	21.05%	5.26%
BsBGLU8	281	31,879.30	5.51	−0.122	36.51	33.81%	15.66%	6.76%
BsBGLU9	194	22,009.19	7.78	−0.198	23.75	38.66%	12.37%	8.25%
BsBGLU10	385	43,354.97	5.65	−0.361	30.06	39.74%	18.70%	7.01%
BsBGLU12	205	23,452.36	5.48	−0.567	33.24	31.71%	13.66%	7.80%
BsBGLU13	516	58,845.44	5.77	−0.377	35.72	39.15%	17.25%	7.17%
BsBGLU14	140	15,644.62	6.28	−0.414	27.40	37.14%	17.86%	9.29%
BsBGLU15	505	57,173.65	6.47	−0.286	34.93	40.40%	16.83%	7.13%
BsBGLU17	52	6021.87	9.78	−0.758	37.74	21.15%	34.62%	7.69%
BsBGLU19	179	20,369.14	5.94	−0.191	32.11	34.08%	22.91%	9.50%
BsBGLU21	512	58,360.99	5.78	−0.351	34.38	38.48%	16.21%	8.01%
BsBGLU22	393	45,329.61	6.76	−0.391	33.27	36.13%	19.08%	6.87%
BsBGLU23	78	9160.42	8.89	−0.362	19.11	52.56%	17.95%	10.26%
BsBGLU24	511	57,984.68	8.10	−0.273	30.84	36.99%	17.81%	6.46%
BsBGLU25	271	30,679.35	5.23	−0.355	28.29	38.38%	16.24%	10.33%
BsBGLU27	244	27,558.07	6.23	−0.268	26.30	40.16%	18.44%	6.15%
BsBGLU28	402	45,398.52	6.92	−0.117	27.73	38.31%	18.41%	5.47%
BsBGLU29	447	50,939.02	5.79	−0.303	27.51	39.60%	15.44%	5.82%
BsBGLU30	350	40,122.47	6.08	−0.518	45.84	32.00%	14.29%	5.71%

## Data Availability

The data presented in the study are deposited in the NCBI repository, accession number PRJNA1009214.

## Supplementary Materials

The following supporting information can be downloaded at https://www.mdpi.com/article/10.3390/ijms252313191/s1.
