# Supplementary material for: Unveiling the Role of β-Glucosidase Genes in Bletilla striata’s Secondary Metabolism: A Genome-Wide Analysis
Source: Int J Mol Sci. 2024 Dec 8;25(23):13191. doi: 10.3390/ijms252313191 (PMC11642090; doi:10.3390/ijms252313191)
Supplement: Supplementary file 1 [file ijms-25-13191-s001.zip › Table S1.pdf]

Table S1 The information of EST-SSR primers

Gene name	Repeat	Forward primer	Reverse primer	Target band size (bp)
BSBGLU1	(TA)7	TGAACATATACGATGTGCTCAACA	TCACCGGTGCTCTTATCAGC	167
BSBGLU8	(TC)16	TCATGGCCTTAGTCTTGAATTTTTG	TTGTTGTTTACAAATGCCCGTG	201
BSBGLU10	(A)11	TCGGGACATCAATGCAGACA	TCCTTGAAGACTGCAACATGT	137
BSBGLU23	(TTA)5	AGCATCATCTTCTTCTTCATCAAAA	CCCGTGATGCAGCCAATCTA	229
BSBGLU25	(TC)6	GGCACTGAACCTTGACTCGA	CGACTTGTGGATATTTATCTCTTTCGT	205
BSWRKY30	(AT)7	TGTAACCAGCAGCAACCCTT	GGAGGTTTGAGAAAATCCACGG	179
