# Supplementary material for: Unveiling the Role of β-Glucosidase Genes in Bletilla striata’s Secondary Metabolism: A Genome-Wide Analysis
Source: Int J Mol Sci. 2024 Dec 8;25(23):13191. doi: 10.3390/ijms252313191 (PMC11642090; doi:10.3390/ijms252313191)
Supplement: Supplementary file 1 [file ijms-25-13191-s001.zip › Table S2.pdf]

Table S2 The information of qPCR primers

Gene	Primer	Tm	GC%
BsBGLU_8F	TCATGGCCTTAGTCTTGAATTTTTG	60.00	55.00
BsBGLU_8R	TTGTTGTTTACAAATGCCCGTG	60.00	55.00
BsBGLU_9F	GGCACATTCCACTGCTGTTG	60.00	55.00
BsBGLU_9R	GCGTGCTTCCAATGAAACCA	59.70	50.00
BsBGLU_10F	TCGGGACATCAATGCAGACA	60.00	55.00
BsBGLU_10R	TCCTTGAAGACTGCAACATGT	60.00	55.00
BsBGLU_12F	CCCCTCTGCTCTCTCTTCCT	60.00	60.00
BsBGLU_12R	GGGCTCAAGAATCCTGTGCT	60.00	55.00
BsBGLU_14F	CACAGAAGGGCTCCTGTACA	59.00	55.00
BsBGLU_14R	GGATTGATAGCTCCTCGTCCA	59.00	52.40
BsBGLU_25F	GGCACTGAACCTTGACTCGA	60.00	55.00
BsBGLU_25R	CGACTTGTGGATATTTATCTCTTTCGT	60.00	60.00
actinF	AATCCCAAGGCAAACAGA	60.00	55.00
actinR	CACCATCACCAGAATCCAG	60.00	55.00
18SrRNA-F	CTACGTCCCTGCCCTTTGTACA	60.00	55.00
18SrRNA-R	ACACTTCACCGGACCATTCAA	60.00	55.00
