# Supplementary figures and images for: Unveiling the Role of β-Glucosidase Genes in Bletilla striata’s Secondary Metabolism: A Genome-Wide Analysis
Source: Int J Mol Sci. 2024 Dec 8;25(23):13191. doi: 10.3390/ijms252313191 (PMC11642090; doi:10.3390/ijms252313191)

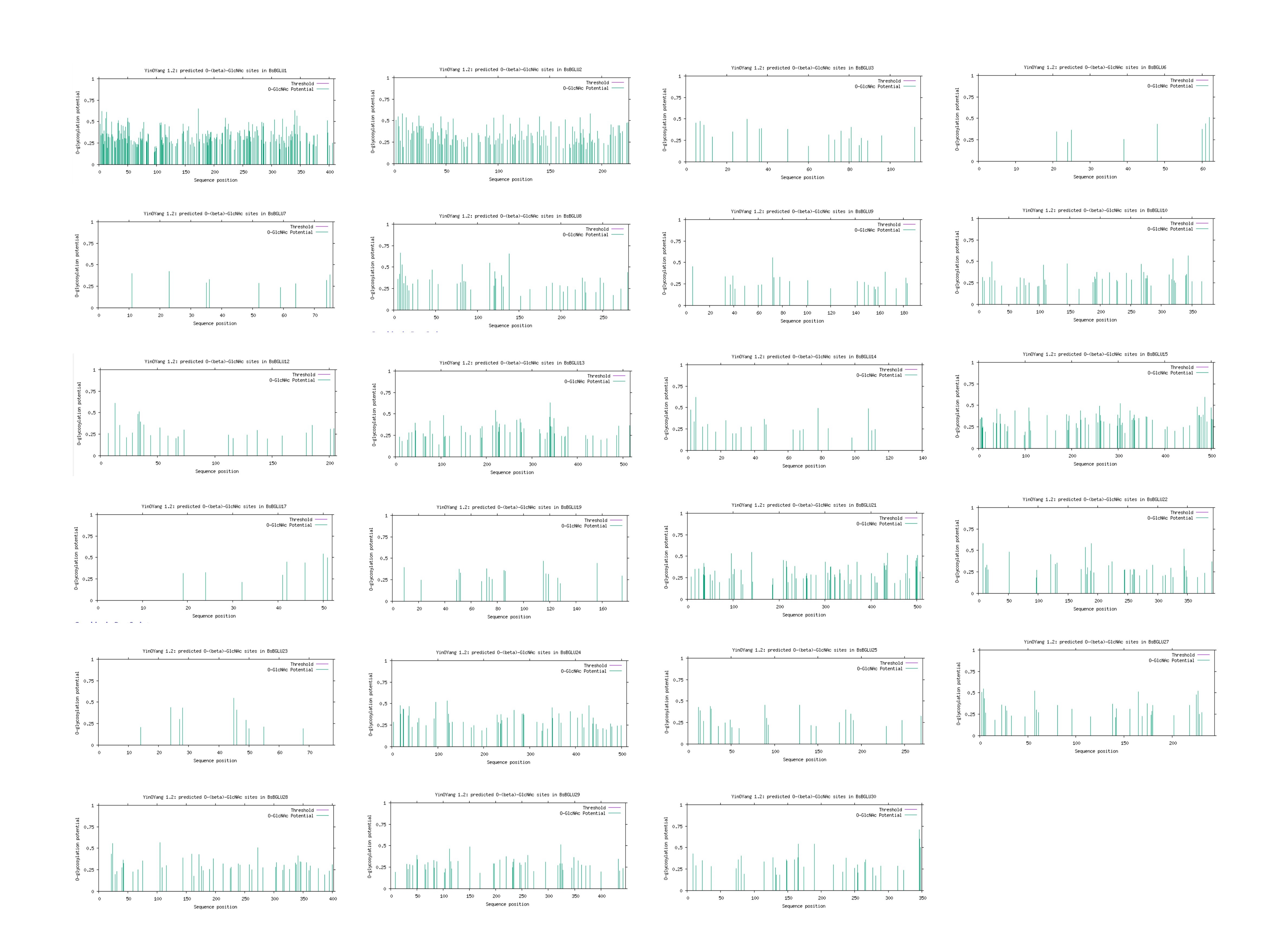

Supplement: Supplementary file 1 [file ijms-25-13191-s001.zip › Figure S1.jpg]
